# The Effect of Internet Use on Older Adults’ Executive Function: The Chain Mediation Effect of Social Participation and Loneliness

**DOI:** 10.3390/healthcare14081071

**Published:** 2026-04-17

**Authors:** Jing Xu, Na Li, Yu Jian, Xin Yang, Xianwen Li

**Affiliations:** 1 School of Nursing, Nanjing Medical University, Nanjing 211166, China; 202011125811074@zcmu.edu.cn; 2 The Second Affiliated Hospital, Nanjing Medical University, Nanjing 210011, China; 13205577289@163.com; 3 The First Affiliated Hospital, Nanjing Medical University, Nanjing 210029, China; yujian4761@jsph.org.cn

**Keywords:** older adults, internet use, executive function, social participation, loneliness

## Abstract

**Background/Objectives**: This study aimed to explore the association between internet use and executive function among older adults and the mediating role of social participation and loneliness in internet use and executive function. **Methods**: A cross-sectional study was conducted among 439 community-dwelling older adults (≥60 years) in Nanjing, China, from September to December 2022. Participants were selected using simple random sampling and assessed with four standardized instruments: the Internet Use Questionnaire, the Social Participation Capacity Assessment, the six-item UCLA Loneliness Scale (ULS-6), and the Behavior Rating Inventory of Executive Function-Adult Version (BRIEF-A). Data were analyzed with the SPSS 21.0 software for descriptive statistics and correlation analysis and the AMOS 23.0 software for structural equation modeling to test the chain mediation effects. Model fit was evaluated using Root Mean Square Error of Approximation (RMSEA), Comparative Fit Index (CFI), Tucker–Lewis Index (TLI), and Weighted Root Mean Square (WRMR), with bootstrap resampling for indirect effect estimation. **Results**: The results showed that internet use was positively correlated with loneliness (r = 0.203, *p* < 0.01), social participation impairment (r = 0.193, *p* < 0.01), and executive function (r = 0.420, *p* < 0.01). Structural equation modeling showed that greater internet use was significantly associated with poorer executive function (β = 0.306, *p* < 0.01). These associations were partially explained by pathways involving social participation and loneliness through three indirect pathways: internet use via social participation (indirect effect = 0.087, 18.3% of the total effect); internet use via loneliness (indirect effect = 0.049, 10.3%); and internet use via social participation and then loneliness in sequence (indirect effect = 0.035, 7.1%). **Conclusions**: In community-dwelling older adults, more frequent internet use was associated with greater executive function impairment through mechanisms involving reduced social participation and increased loneliness. Therefore, there is a need to limit excessive internet use while promoting social participation and reducing isolation, which can have the greatest benefits for executive functioning in older adults.

## 1. Introduction

With the rapid increase in the proportion of the global older adult populations, the cognitive health issues of older adults have increasingly become a prominent focus of attention. In this context, investigating the executive function of older adults has assumed particular significance. The number of older adult netizens aged 60 and above in China has reached 119 million, accounting for 11.5% of the total netizens [1]. Previous studies have indicated that subjective (mild) decline in executive function can be considered a precursor to dementia, significantly impairing the health-related quality of life in older adults [2].

The internet is used for various activities, the popular ones being sending and receiving e-mails, finding information, reading online news, participating in social networks and making phone or video calls. Currently, researchers primarily measure the extent of internet usage in terms of frequency [2], intensity [3], and addiction [4]. Results of a cross-sectional exploratory observational study targeting older adults [5] found that previous internet search experience may alter the brain’s ability to respond in neural circuits that control decision making and complex reasoning in middle-aged and older adults individual. However, a study pointed out that internet usage may lead to a decline in individuals’ cognitive abilities, particularly with negative effects on executive function [6]. The ongoing debate regarding the impact of internet use on executive functioning in older adults underscores the need for continued research in this area.

The impact of internet use on executive functioning in older adults is still controversial, and further research on the mechanisms of its impact is needed to address this inconsistency. Executive function is considered a fundamental cognitive skill and plays a crucial role in independent functioning. Research has indicated a close relationship between executive function and the risk of falls, particularly severe falls, related to age [7,8]. Additionally, executive function impairment has been identified as a robust predictor of mortality, frailty, and disability [9,10,11]. Analyzing factors affecting executive functioning is a prerequisite for promoting health in older adults. There are various factors that have been extensively discussed that affect executive function in older adults, including demographic characteristics [12], exergaming [13], socio-psychological factors, and lifestyle [14]. However, previous research has focused less on the potential effects of internet use on executive function in older adults in general. Therefore, with the explosive popularity of the internet, it is crucial to consider the potential influence of internet usage on executive function.

A lack of social contact is also one of the main causes of loneliness among older adults. There is a correlation between internet usage and the reduction in loneliness in older adults [15]. Elderly individuals often feel lonely due to various factors such as low self-efficacy, social anxiety, a lack of social support, and depression. As a result, they may not actively seek social interactions, further exacerbating their sense of loneliness [16]. It is worth noting that analyzing the social participation of older adults can help improve their loneliness and promote physical and mental health. There is a substantial body of evidence indicating that social participation serves as a protective factor against loneliness [13,17].

**Figure 1:**
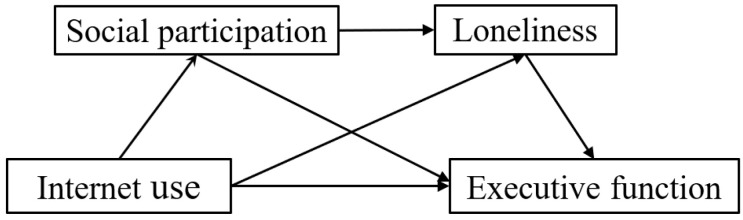
Hypothetical modeling of the four indicators.

Although a prior work has separately linked internet use, social participation, loneliness, and executive function among older adults, no study has simultaneously examined these constructs within a single, sequential framework. Integrating activity theory and cognitive reserve theory, we advance a chain-mediation model that positions social participation and loneliness as successive mechanisms through which internet use influences executive function. Drawing on activity theory’s proposition that role continuity protects against disengagement, we first hypothesize that higher levels of internet use will be negatively associated with older adults’ real-world social participation (H1). Consistent with cognitive reserve theory’s emphasis on social engagement as a protective resource, we expect that diminished social participation will, in turn, predict elevated feelings of loneliness (H2). Extending this to stress-vulnerability mechanisms, we posit that greater loneliness will be related to poorer executive function (H3). Integrating these theoretical propositions, we hypothesize that the association between internet use and executive function will be significantly mediated by the ordered sequence of social participation and loneliness (H4). The hypothesized model is displayed in Figure 1.

## 2. Materials and Methods

### 2.1. Participants and Procedure

A retrospective cross-sectional research design was adopted in this study, and the data of relevant indicators were collected through questionnaires. The inclusion criteria for the study subjects were as follows: (a) aged 60 years and above; (b) without severe cognitive impairment or mental illness; (c) with previous experience of using internet; and (d) agreement to participate voluntarily in this study. We excluded study participants if they have communication disorders. The sampling frame comprised older adults registered in the chronic disease management system of these centers. Between 1 September and 15 December 2022, 439 community-dwelling older adults (≥60 years) in Nanjing, China, completed face-to-face surveys (response rate: 97.6%; 6 declined and 5 excluded for communication/cognitive impairment). Participants were selected via simple random sampling from community health registries. The investigators received unified training and were required to provide necessary explanations to older adults who had difficulty understanding the questionnaire.

All patients signed informed consent. This study was approved by the Ethics Committee of the Second Affiliated Hospital of Nanjing Medical University, Nanjing, Jiangsu Province, China (ethical approval number: KY-2022-004-01).

(1) $$N={\left(\frac{Z_{1-\alpha /2}}{\delta }\right)}^{2}\times p\times \left(1-p\right)$$

*N* represents the sample size of each group, and Z((1−α)/2) is 1.96 in this study; δ represents the permissible error, which is taken as 0.10 in this study; according to the survey, the internet usage rate of Chinese seniors over 60 years of age is 48.6% [18], and *p* is taken as 0.486; substituting the formula yields the number of study subjects to be included as 422.

To ensure validity, a three-stage protocol was followed. First, trained interviewers provided on-site clarification of every item prior to administration. Second, upon completion, each questionnaire was immediately inspected for completeness and logical consistency. Third, two researchers independently re-examined all questionnaires before data entry. A total of 450 questionnaires were distributed; after excluding 11 that had >10% missing or logically inconsistent responses, 439 valid questionnaires remained (validity rate = 97.6%). The final sample comprised 242 males and 197 females.

### 2.2. Measures

#### 2.2.1. Demographic Characteristics

A questionnaire was used to collect demographic information on the study participants, including age, gender, educational level, marital status, residency, number of children, comorbid chronic disease, income (RMB), sleep problems, and exercise frequency, and the on-site collection of signed research consent forms from participants was conducted.

#### 2.2.2. Behavior Rating Inventory of Executive Function-Adult Version, BRIEF-A

BRIEF-A has been widely used internationally to measure participants’ executive function. It was translated and introduced by Shankar [19]. The questionnaire consists of 75 items (including five polygraph questions) and uses a 1–3 point score, with 1 for “never”, 2 for “sometimes” and 3 for “often”. After the participants completed the questionnaire, scores for 70 items were calculated to obtain a Global Executive Composite (GEC), which is derived from two index scores: the Behavioral Regulation Index (BRI) and the Metacognitive Index (MI). Among them, BRI was composed of four factors: inhibition, conversion, self-monitoring and emotional control. MI includes five factors: initiation, working memory, planning, material organization, and task monitoring. The higher the factor or total score, the worse the participant’s executive function. Previous studies have shown high reliability and validity of this scale. In this study, Cronbach’s α coefficient was 0.913 for the total scale [20].

#### 2.2.3. Chinese Version of the ULS-8 Loneliness Form

The ULS-8 Loneliness form is one of the most widely used loneliness measurement tools internationally. In order to adapt to the Chinese context, the ULS-8 was sinicized and revised, and two entries were deleted [21]. The ULS-6 form was finally used in this study, which consists of 6 items, each rated on a 5-point Likert scale (1 = never to 5 = always). The total score ranges from 6 to 30, with higher scores indicating greater loneliness. Subjects responded according to their feelings in recent times. The Cronbach’s α coefficient in this study was 0.935. Loneliness is defined as an individual’s subjective experience on an emotional and social level, reflecting the gap between the desire for social connection and the lack of actual social connection.

#### 2.2.4. Social Participation Capacity Assessment

As a comprehensive assessment tool of older adults, the social participation assessment questionnaire has been widely used in clinical practice [22]. The social participation assessment questionnaire—after local cultural adaptation and item reduction—was used as a comprehensive tool to evaluate older adult respondents. The revised version retains five sub-dimensions: life ability, work ability, time/space orientation, character orientation and social communication ability. Orientation subscales (time/space and person orientation) may conceptually overlap with executive function domains such as spatial working memory and attentional control. However, the form has been widely used in Chinese older adult populations. The form adopts the Likert 4-point scoring method with a total score of 0–20 points, divided into four levels: a score of 0–2 for level 0 (the ability is intact), a score of 3–7 for level 1 (mild damage), a score of 8–13 for level 2 (moderate damage), and a score of 14–20 for level 3 (severe damage). The higher the score, the more impaired the ability to participate in society. The Cronbach’s α coefficient in this study was 0.802. Social participation is defined as the extent to which an individual is actively involved in a variety of social and societal activities. This includes, but is not limited to, interactions with friends, family and neighbors, participation in community organizations or groups, and involvement in public affairs and activities.

#### 2.2.5. Internet Use

The Internet Use Questionnaire was developed on the basis of the internet-use module of the CHARLS 2018 survey. After cultural and linguistic adaptation for the local older adult population, five items were retained: information searching, sending/receiving e-mails or WeChat messages, online shopping and bill payment, social media interaction, and a reverse-coded item on perceived internet dependence. Each item is rated on a 5-point Likert scale (1 = never to 5 = always); item 3 is reverse-scored. The total score ranges from 5 to 25, with higher scores indicating more frequent and diverse internet use [23]. The Cronbach’s α coefficient in this study was 0.800. Internet use was defined as individuals browsing websites, using online applications, engaging in social media, or performing other internet-related activities via a mobile device, laptop, or computer. This includes behaviors that individuals engage in independently or in collaboration with others, either during or outside of work hours.

### 2.3. Data Analysis

Statistical analysis was performed using SPSS 26.0 (IBM Corp, Armonk, NY, USA). Categorical and continuous data were described using descriptive statistics (means, standard deviations, frequencies, and percentages). Binary correlation analysis was used for the four variables. Normally distributed continuous variables were compared between two groups with an independent-samples *t*-test; for three or more groups, one-way analysis of variance (ANOVA) was applied.

In addition, structural equation modeling (SEM) was conducted using AMOS 23.0. Path analysis (PA) was used to evaluate the mediation model. Model fit was evaluated using several fit indices, i.e., (1) the chi-square (χ^2^) test of model fit, (2) the Root Mean Square Error of Approximation (RMSEA), (3) the Comparative Fit Index (CFI), (4) the Tucker–Lewis Index (TLI), and (5) Weighted Root Mean Square (WRMR), due to the presence of binary data. An RMSEA value of less than 0.06, CFI at or above 0.90, TLI at 0.90 or higher, and WRMR value that was below 1.00 indicate a relatively good fit [24]. The Monte Carlo method was used to test the significance of indirect mediation effects by generating 95% confidence intervals through 5000 bootstrap resampling iterations. If the confidence interval for an indirect effect did not contain zero, the mediation effect was considered statistically significant.

## 3. Results

The basic information of the subjects in the survey is shown in Table 1. A total of 439 subjects were enrolled in this study, of which 242 (55.1%) were males and 197 (44.9%) were females. The mean age of participants was 67.4 ± 5.8 years (range: 60–89 years). There were 342 cases (77.9%) in the age group of 60–69 years. Educational level was secondary school in 231 cases (52.6%). All the study subjects were married. There were 23 cases (5.2%) living alone. A total of 206 cases (46.9%) had two children. Comorbid chronic diseases were present in 223 cases (50.8%). The average monthly income was between RMB 1001 and 3000 in another 166 cases (37.8%). Sleep problems were present in 129 cases (29.4%). The frequency of exercise was more than three times a week in 194 cases (44.2%). Mean executive-function scores differed significantly across gender, age, educational levels, residency, number of children, income, sleep problems, and exercise frequency (all *p* < 0.05).

**Figure 2:**
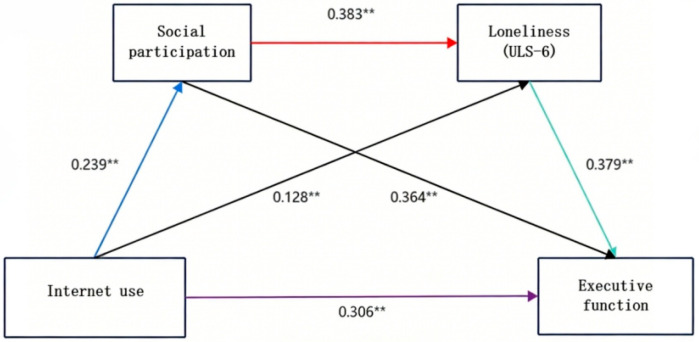
The diagram of the chain mediator of social participation and loneliness. Note: All coefficients are standardized values, and ** indicates *p* < 0.01. Higher scores on social participation and executive function scales indicate greater impairment.

Table 2 reports descriptive statistics and correlative statistics among variables. Internet use was positively correlated with loneliness (r = 0.203, *p* < 0.01), social participation (r = 0.193, *p* < 0.01), and executive function (r = 0.420, *p* < 0.01). Notably, higher scores on the social participation form indicate greater impairment in participation ability. Because there was a strong link between internet use, loneliness, social participation and executive function among older adults, the conditions for the mediating effect test were met. Therefore, taking internet use as the independent variable, loneliness and social participation as the mediating variables, and executive function as the dependent variable, the structural equation was established, and the initial model fitting degree was good: 2/df = 1.850, RMSEA = 0.044, IFI = 0.950, TLI = 0.945, and CFI = 0.950. As shown in Figure 2, internet use showed a significant positive association with executive functioning (β = 0.306, *p* < 0.001), which was further amplified by two parallel mediating chains: (i) the path coefficients of “internet use → social participation impairment → executive functioning” were 0.239 and 0.364, respectively; (ii) the path coefficients of “internet use → loneliness → executive functioning” were 0.128 and 0.379, respectively. In addition, social participation also positively predicted loneliness (β = 0.383, *p* < 0.001), forming a chain of serial mediators.

The bootstrap method, which can be estimated by the confidence interval, was further selected for testing, and the 95% confidence interval was calculated by repeated sampling 5000 times, and if the obtained confidence interval did not contain 0, the corresponding mediation effect was significant. The results suggest that social participation and loneliness play multiple mediating roles between internet use and executive function in older adults. The total mediation effect is composed of indirect effects produced by three pathways, and the indirect effects of internet use through the social participation of older adults, loneliness in older adults, and social participation–loneliness mediation chain on the executive function of older adults are significant, which again indicates that all intermediary paths are established. The effect values and effect magnitudes of each pathway are shown in Table 3 below.

## 4. Discussion

The results of this study indicated that greater internet use was associated with higher levels of social participation impairment, poorer executive function, and greater loneliness. Social participation impairment was positively associated with both loneliness and executive function impairment, and loneliness was positively associated with poorer executive function. Internet use showed significant associations with executive function through the pathways involving social participation, loneliness, and the sequential social participation–loneliness mediation chain (*p* < 0.05). The associations of social participation, loneliness, and the mediating chain with executive function in older adults were significant (*p* < 0.05). In brief, social participation and loneliness appear to function as multiple mediators in the relationship between internet use and executive function, with potentially three mediation pathways.

Our results indicate that there is an inverse relationship between internet usage and executive function among older adults. Currently, there is still controversy about the relationship between internet use and executive function, which highlights the importance of current research. A study in the United States [25] found that older adults can increase executive function through internet use and increased daily social contact. Maria [26] found that internet use in older adults experiencing homelessness has a positive effect on executive function. This is inconsistent with our findings, which may be attributed to the fact that we studied older adult individuals in socially supported urban communities where all participants were married and had access to stable family support, potentially amplifying the negative effects of excessive internet use on executive function [27]. Our findings imply that promoting strategies to reduce internet usage among older adult individuals, particularly those with limited social support or living alone, can effectively help counteract the decline in executive function. In the future, further longitudinal study designs are needed to confirm the relationship between internet usage intensity and executive function.

Social interaction is one of the most important factors for older adults to use internet. For older adult individuals, social participation is crucial for adapting to society, improving their quality of life, and enhancing psychological well-being. It can be defined as an individual’s involvement in activities that interact with others in the social or community context, expressing interpersonal communication beyond the confines of home [28]. Contrary to expectations that internet use would enrich social participation [29], our findings showed that greater internet use was associated with higher levels of social participation impairment. This may suggest that excessive internet use displaces real-world social activities, or that individuals with pre-existing social participation difficulties turn to the internet as an alternative. Research has shown that better social participation can promote cognitive improvement in older adults [30]. The long-term lack of social participation can lead to changes in brain structure and executive function in older adults, increasing the risk of dementia, while active social participation can slow down or inhibit cognitive decline [13]. According to cognitive reserve theory, the greater the cognitive reserve, the stronger the cognitive risk resistance and the better the cognitive task performance [31]. Social participation is an important protective factor of cognitive reserve. The literature based on the theory of resocialization of old age and activity theory believes that, even if the individual enters the old age stage of their life cycle and their physiological and psychological qualities may weaken, they should still maintain an active state and social participation, continuously learn to adapt to society, maintain health and realize value. The use of the internet has greatly enriched the lives of older adults. Contrary to our findings, some studies have shown that older adults can expand their social networks and ultimately enhance their social adaptability and participation through activities such as e-commerce and online entertainment [32].

In addition, the mediating effect of loneliness on internet use and executive function in older adults is also significant. The indirect path “internet use—loneliness—executive function” explained 10.3% of the total effect. Online social media is a tool and platform used by people to share opinions and experiences. Internet usage can help alleviate loneliness by expanding or maintaining external connections that compensate for the lack of substantive social interactions. Loneliness functions as a key psychological mediator because sustained social disconnection triggers chronic stress responses that are neurotoxic to prefrontal–hippocampal circuits critical for executive control. Although this mechanistic pathway has been consistently validated in adolescent cohorts, its existence and underlying characteristics in older adult populations await further empirical investigation [33]. Our study showed that loneliness scores significantly and positively predicted executive functioning scores (β = 0.379, *p* < 0.001), and higher levels of loneliness were associated with more impaired executive functioning. This is consistent with the results of the study of Lara and Ren [34,35]. Loneliness is a common negative psychological emotion in older adults. As a classic negative emotion, loneliness has common neurophysiological mechanisms with depression, which is confirmed to damage the hippocampus by raising glucocorticoid levels, triggering cerebral inflammation and vascular diseases [36]. Loneliness may exert a detrimental effect on older adults’ executive function through this similar mechanism [37]. Loneliness has a close relationship with executive functions [38]. A meta-analysis showed that long-term loneliness significantly increases the risk of Alzheimer’s disease in older adults [39]. Furthermore, research also found associations between loneliness and decreased cognitive flexibility and decision-making abilities [40,41]. Individuals with higher levels of loneliness may face greater difficulties in adjusting attention, switching tasks, or making rational decisions. In addition, attitudes toward the internet and its use may be related to the cognition and behavior of older adults, and positive attitudes may motivate older adults to adopt the internet more positively and thus enjoy its benefits, including information access, social connectivity, and cognitive stimulation [42]. It is important to note that the negative association between internet use and executive function observed in this study may be specific to our sample characteristics. Previous studies have demonstrated the positive effects of internet use on cognitive function among vulnerable populations with limited social support, such as older adults experiencing homelessness or those living alone in urban settings [43]. For these populations, internet use serves as a critical tool for maintaining social connections and accessing health information, thereby compensating for structural social deficits. In contrast, our study participants were predominantly married community-dwelling older adults with stable family support systems. For such individuals, excessive internet use may represent a displacement of real-world cognitive stimulation rather than a compensatory mechanism, potentially explaining the divergent findings across different socioeconomic contexts.

A key finding of the present study is that social participation and loneliness sequentially mediate the relationship between internet use and executive function; however, the indirect effect attributable to the social participation pathway accounts for only 7.1% of the total effect. The modest mediating role of social participation can be interpreted through activity theory. According to activity theory, encouraging the social participation of older adults can improve loneliness and depression caused by disconnection with social development and disruption of social roles [44]. Older adults with high levels of loneliness are more likely to experience executive function loss [45]. This is because loneliness may affect cognitive decline through an exaggerated response to stress, leading to neurodegeneration in the hippocampus or reduced connectivity within the prefrontal cortex, which in turn affects executive function [46]. Collectively, these mechanisms limit the extent to which the effect can be attributed to the social participation pathway. Therefore, improving the level of social participation can prevent negative emotions such as loneliness and thereby protect executive function in a certain way [47]. Furthermore, this study found a positive correlation between internet use and loneliness. This indicates a potential compensation mechanism whereby lonely individuals may use the internet as a substitute for offline social interaction. However, our cross-sectional design precludes definitive causal conclusions regarding whether internet use exacerbates loneliness or merely serves as a coping strategy for socially isolated individuals. Longitudinal research is warranted to disentangle these temporal relationships.

Our model shows that internet use affects the executive function of older adults through the independent and combined effects of two mediating variables, social participation and loneliness, and further explains the specific mechanism of the impact of internet use on the executive function. Internet use is a predictor of executive function, and social participation and loneliness can be mediated alone or as a chain mediator between internet use and executive function in older adults. Our findings have positive implications for the protection of executive function in older adults. Based on the above conclusions, this study puts forward the following countermeasures and suggestions: firstly, improve the internet penetration rate of older adults, actively encourage relevant network products to be age-appropriate and barrier-free, and encourage communities to carry out network software operation training to assist older adults to master basic communication and mobile payment technologies; secondly, encourage older adults to actively participate in social activities, and provide employment consultation, employment introduction and employment services for older adults to re-employment; and finally, reducing loneliness is an important focus for protecting their executive function, and for older adults who are unable to participate in digital networks, intervention in loneliness can also play a role in protecting their executive function.

This study has some limitations. First of all, this study mainly relies on a questionnaire as the main research method. In older adults, there may be a recall bias, which affects the accuracy of the results. Secondly, our cross-sectional research cannot explain the profound relationship between the variables. The cross-sectional design precludes causal inferences regarding the direction of associations. Longitudinal and qualitative research methods may make up for this. In addition, social participation and loneliness play an important role in the process of changing executive function in older adults. However, other internal personal characteristics or external factors may also affect executive function in older adults. Multiple sociodemographic factors showed significant bivariate associations with executive function. Future longitudinal studies should adjust for these potential confounders to isolate the independent effect of internet use. Thirdly, the social participation form includes orientation subscales that may conceptually overlap with executive function domains, potentially inflating observed associations between these constructs. The internet use measure combined diverse behaviors, precluding conclusions about which specific usage patterns may be beneficial or harmful. Finally, the sample was moderate-sized and single-site, drawn from a single urban community in Nanjing, China, and exhibited demographic homogeneity. These findings may not generalize to rural populations, other cultural contexts, or older adults with different marital and socioeconomic statuses.

## 5. Conclusions

This study found that a higher frequency of internet use among well-supported community-dwelling older adults was associated with poorer executive function, and this relationship was partially mediated by social participation and loneliness. Specifically, internet use was associated with greater impairment in real-world social participation and exacerbated loneliness, thereby impairing executive function. These findings suggest that, within this specific sociocultural context, the moderate restriction of internet use, coupled with the promotion of social participation and alleviation of loneliness, may confer protective benefits for executive functioning in older adults. These findings generate hypotheses for future longitudinal research examining (a) which specific internet use patterns relate to cognitive outcomes and (b) whether supporting real-world social participation and addressing loneliness may help maintain executive function in aging.

## Figures and Tables

**Table 1:** Sociodemographic characteristics and executive function scores of participants (*N* = 439).

Characteristics	Category	*N* (%)	Mean (SD)	*t*/F	*p*
Gender	male	242 (55.1)	117.74 (15.39)	2.848	0.005
	female	197 (44.9)	122.45 (19.23)		
Age (year)	60–69	342 (77.9)	117.65 (15.90)	19.219	0.000
	70–79	78 (17.8)	124.77 (16.87)		
	above 80	19 (4.3)	139.26 (27.09)		
Education level	elementary school	123 (28.0)	126.85 (19.25)	12.413	0.000
	secondary school	231 (52.6)	117.59 (15.49)		
	high school	68 (15.5)	118.21 (17.38)		
	undergraduate and above	17 (3.9)	106.53 (7.99)		
Marital status	married	439 (100. 0)	119.89 (17.39)	-	-
Residency	lives alone	23 (5.2)	131.57 (24.87)	3.362	0.001
	does not live alone	416 (94.8)	119.20 (16.66)		
Number of children	0–1	134 (30.6)	116.27 (16.04)	5.436	0.001
	2	206 (46.9)	119.77 (16.52)		
	≥3	99 (22.6)	125.07 (19.59)		
Comorbid chronic disease	yes	223 (50.8)	120.82 (19.04)	1.183	0.238
	no	216 (49.2)	118.86 (15.41)		
Income (RMB) ^a^	≤1000	72 (16.4)	124.21 (16.88)	4.168	0.006
	1001–3000	166 (37.8)	120.72 (18.33)		
	3001–5000	125 (28.5)	119.44 (16.50)		
	≥5000	76 (17.3)	114.50 (15.89)		
Sleep problems ^b^	yes	129 (29.4)	123.38 (20.71)	2.767	0.006
	no	310 (70.6)	118.39 (15.57)		
Exercise frequency	none	81 (18.5)	126.09 (21.93)	5.559	0.001
	once a week	54 (12.3)	115.44 (12.28)		
	two–three times a week	110 (25.1)	117.39 (15.53)		
	more than three times a week	194 (44.2)	119.87 (16.79)		

^a^ Average monthly income; ^b^ poor sleep on more than 3 days of the week for more than 3 months.

**Table 2:** Pearson correlation analysis among different variables (*N* = 439).

Variable	M	SD	(1)	(2)	(3)	(4)
(1) Internet use	9.412	3.660	1			
(2) Loneliness	12.621	5.872	0.203 **	1		
(3) Social participation	4.150	2.659	0.193 **	0.352 **	1	
(4) Executive function	119.852	17.365	0.420 **	0.508 **	0.447 **	1

** *p* < 0.01.

**Table 3:** Chain mediation analysis test between social participation and loneliness in internet use and executive function (*N* = 439).

Path	Meditation Effect (95% CI)	*p*	Proportion of Effect
Indirect effect			
Internet use ^a^ → social participation ^b^ → executive function ^c^	0.087 (0.047, 0.146)	0.001	18.3%
Internet use → loneliness ^d^ → executive function	0.049 (0.002, 0.098)	0.049	10.3%
Internet use → social participation → loneliness → executive function	0.035 (0.016, 0.059)	0.001	7.1%
Direct effect			
Internet use → executive function	0.306 (0.201, 0.400)	0.001	64.3%
Total effect			
Internet use → executive function	0.476 (0.350, 0.584)	0.001	100.0%

^a^ Internet Use Questionnaire; ^b^ Social Participation Capacity Assessment; ^c^ loneliness (ULS-6); ^d^ Behavior Rating Inventory of Executive Function-Adult Version.

## Data Availability

The data are not publicly available due to the protection of privacy and confidential information of the research participants. The data that support the findings of this study are available from the corresponding author upon reasonable request.
